# Minocycline ameliorates CNS autoimmunity through restraint of CD4^+^CD11bc^+^ cells

**DOI:** 10.3389/fimmu.2026.1823730

**Published:** 2026-09-03

**Authors:** Zorana Milosavljević, Milica Lazarević, Goran Stegnjaić, Neda Nikolovski, Ksenija Mirković, Dmitri Lodygin, Alexander Flügel, Đorđe Miljković, Suzana Stanisavljević, Bojan Jevtić

**Affiliations:** 1 Department of Immunology, Institute for Biological Research “Siniša Stanković” - National Institute of the Republic of Serbia, University of Belgrade, Belgrade, Serbia; 2 Institute for Neuroimmunology and Multiple Sclerosis Research, University Medical Center Göttingen, Göttingen, Germany

**Keywords:** macrophage, microglia, minocycline, nitric oxide, nuclear factor erythroid 2-related factor 2, reactive oxygen species

## Abstract

Minocycline is a tetracycline antibiotic with profound anti-inflammatory activity directed primarily against microglial cells and macrophages. Activation of nuclear factor erythroid 2-related factor 2 (Nrf2) is considered as an important target of minocycline. This drug has been considered a promising therapeutic for multiple sclerosis, an autoimmune disorder of the central nervous system (CNS). Still, its effect on CNS autoimmunity is not entirely understood. The aim of this study was to investigate the potency of short-term minocycline administration in fully developed CNS inflammation. To this end, an animal model for multiple sclerosis, experimental autoimmune encephalomyelitis (EAE), was used and minocycline was applied at the peak of the disease. The treatment led to immediate reduction of EAE clinical signs, and also to reduction of overall EAE clinical course severity. The effect of minocycline at the peak of EAE was paralleled with inhibited NO and reactive oxygen species (ROS) production by immune cells in CNS. Interestingly, the reduction was specific for CD4^+^CD11bc^+^ cells, as it was not observed in CD8^+^CD11bc^+^ cells or neutrophils. Minocycline efficiently reduced NO and ROS generation in macrophages *in vitro*. Surprisingly, this effect was not abolished in macrophages obtained from Nrf2 knockout mice. Further, beneficial effects of minocycline in EAE were also observed in Nrf2 knockout mice, thus corroborating the notion that Nrf2 activation is not the decisive way of anti-encephalitogenic effects of the drug. Taken together, these data indicate that beneficial effects of minocycline in EAE is primarily based on its ability to limit the generation of ROS and NO in CNS CD4^+^CD11bc^+^ cells and that its overall effect on EAE is Nrf2-independent. Our results substantiate the idea that minocycline is a valid candidate for further multiple sclerosis-related research and point to CD4^+^CD11bc^+^ cells as an important disease causative myeloid cell population in the CNS autoimmunity.

## 1 Introduction

Minocycline is a second generation tetracycline antibiotic that has been suggested as a promising drug for the treatment of multiple sclerosis (1). Microglial cells and macrophages were identified as the major cellular targets of minocycline in studies on animal models of multiple sclerosis, such as experimental autoimmune encephalomyelitis (EAE) and cuprizone-induced remyelination (2–4). Accordingly, numerous *in vitro* studies showed that minocycline potently inhibits microglia/macrophage ability to produce reactive oxygen species (ROS), nitric oxide (NO), and pro-inflammatory cytokines, including interleukin (IL)-1β, IL-6, and tumor necrosis factor (TNF) (5–8). Macrophages and microglia are active players in the pathogenesis of multiple sclerosis and EAE (9), where their ability to produce ample amounts of ROS, NO and inflammatory cytokines makes them the major effectors in the chronic phase of CNS autoimmunity (10). Thus, research of the ability of minocycline to inhibit the effector functions of macrophages and microglia is of primary interest for its potential use in the treatment of multiple sclerosis.

Minocycline activity in neuroinflammation has been linked to activation of nuclear factor erythroid 2-related factor 2 (Nrf2) pathway. For instance, it was shown that mild stress-induced or sleep deprivation-induced neuroinflammation in mice increases microglial inflammatory properties and decreases Nrf2 level in CNS, while both of these changes were counteracted with minocycline treatment (11, 12). Also, it is suggested that Nrf2 has a crucial role in anti-inflammatory and anti-oxidant effects of minocycline (13, 14). However, direct investigation of the role of Nrf2 in minocycline effects on macrophages/microglia in a neuroinflammatory setting and specifically in EAE is still missing.

In order to fill this gap in knowledge, we investigated the effect of minocycline on EAE and studied the role of Nrf2 in the effects of minocycline on ROS and NO generation in macrophages/microglia *in vivo* and *in vitro* in relation to neuroinflammation. We also investigated EAE course in Nrf2-/- mice to deduce the importance of Nrf2 in minocycline anti-encephalitogenic effects. We were particularly interested to investigate populations of microglia and CNS macrophages expressing CD4 or CD8, as these populations were previously identified as pathogenic in rat EAE (15–18).

## 2 Materials and methods

### 2.1 Animals, models and treatment

Dark Agouti (DA) rats, C57BL/6 mice and Nrf2 knockout (Nrf2^-/-^) mice were bred and maintained in the animal facility of the Institute for Biological Research “Siniša Stanković”. Permissions for the experiments were granted by the Veterinary Administration of the Ministry of Agriculture, Forestry and Water Management, of the Republic of Serbia (No 00432626 2023 14841 002 000 323 022).

Six-month-old female DA were immunized with spinal cord homogenate (SCH), scored and allocated to clinical severity groups, as previously described (18, 19). Minocycline hydrochloride (or doxocycline hyclate (both from Sigma-Aldrich, St Louis, MI) were applied intraperitoneally at 50 mg/kg in 300 µl, once daily from day 11 till day 14 post immunization. Control rats received 300 µl of water as vehicle.

Female wild type and Nrf2^-/-^ C57BL/6 mice (10–12 weeks old) were immunized with myelin oligodendrocyte glycoprotein (MOG)35-55 (SB-PEPTIDE, Saint-Egrève, France), as previously described (20). Complete Freund’s adjuvant (CFA) was obtained by supplementing incomplete Freund’s adjuvant with M. tuberculosis (both from BD Difco, Franklin Lakes, NJ). Pertussis toxin (PT) was from Biotechne, Minneapolis, MN. Minocycline was applied intraperitoneally at 50 mg/kg in 200 µl, once daily from day 12 till day 28 post immunization. Control mice received 200 µl of water as vehicle. Mice were observed daily for clinical scoring: 0 – no motor dysfunctions, 0.5 - limp tip of tail, 1- limp tail, 1.5 - limp tail and slightly wobbly walking, 2 - wobbly walking, hind legs are not spread apart when picked up by the base of the tail, 2.5 - dragging of both hind legs, 3 - complete paralysis of one hind leg, or hind leg paddling, 3.5 - complete paralysis of both hind legs, 4 - complete paralysis of hind legs and partial front leg paralysis, 4.5 – as 4, no movement around the cage, 5 – moribund state or death.

### 2.2 Cells and cell cultures

BV-2 mouse microglial cell line was purchased from Elabscience Biotechnology Inc (Houston, TX). BV2 cells were grown in RPMI-1640 (Capricorn Scientific, Ebsdorfergrund, Germany) supplemented with 10% fetal bovine serum (FBS, Capricorn Scientific) in 96-well plates (5x104/ml). BV2 cells were stimulated with murine IFN-γ (10 ng/ml, Peprotech, London, UK) and lipopolysaccharide (LPS, 10 ng/ml, Sigma-Aldrich, Saint Louis, MO) for 24 hours.

Macrophages were obtained from peritoneal cells, as previously described (21). Peritoneal cells were seeded at 5x10^4^/well in 96-well plates in RPMI-1640 supplemented with 5% FBS. Peritoneal macrophages, obtained after 2 hours as adherent cells, were subsequently stimulated with LPS (10 ng/ml) for 24 hours.

Spinal cord immune cells and brain immune cells were isolated from the immunized rats, as previously described (18, 19). Number of viable cells in cell suspensions was determined after staining with trypan blue dye by cell counting in a hemocytometer. The actual number of cells in specific populations in spinal cord and brain structures was calculated according to the percentages determined by flow cytometry. For spinal cord, numbers are calculated per entire organ, while for brain structures numbers are expressed per g of tissue.

All cell cultures were incubated at 37°C in an atmosphere containing 5% CO2.

### 2.3 Viability assays

Viability of cells was assessed by crystal violet (CV) assay, as described previously (21). The absorbance of dissolved dyes, corresponding to the number of viable cells, was measured in triplicates at 540 nm with a correction at 670 nm using an auto-mated microplate reader Synergy H1 (Agilent Technologies, Santa Clara, CA).

### 2.4 Detection of NO release

DAF-FM staining was used for the determination of NO production. For *in vitro* studies, BV2 cells and peritoneal macrophages were treated with 2 µM DAF-FM diacetate (Thermo Fisher Scientific, Waltham, MA) in the absence or presence of minocyclin for 24 h. Fluorescence intensity (f.i.) was detected with a multiplate reader Synergy H1, using excitation at 510 nm and emission at 540 nm. For ex vivo studies, cells were incubated with 2 µM DAF-FM diacetate for 1 hour, the reaction was stopped by exposure to ice for 10 minutes, and the cells were stained with appropriate antibodies. The fluorescence was acquired via flow cytometry.

### 2.5 Detection of ROS generation

For detection of ROS generation, DHR or H_2_DCF staining was performed. For *in vitro* studies, the cells were incubated in the presence of LPS (10 ng/mL) and the absence or presence of minocyclin for 24 h. Subsequently, cells were incubated with 1 μM DHR (Thermo Fisher Scientific) for 20 min and then stimulated with PMA (100 ng/mL) for 1 hour. For *ex vivo* studies, cells were incubated with 1 μM DHR or H_2_DCF diacetate (Thermo Fisher Scientific) and PMA (100 ng/mL) for 20 minutes. In some experiments, acetovanillone (Acros Organics, Geel, Belgium) was used as inhibitor of NADP oxidase activity. Acetovanillone (100 μM) was applied for 1 hour with the last 20 minutes co-incubation with DHR. The reaction was stopped by exposure to ice for 10 minutes, and the cells were stained with appropriate antibodies. The fluorescence was acquired via flow cytometry and results presented as mean fluorescence intensity (mfi).

### 2.6 Flow cytometry

Cells were stained with the following anti-rat antibodies: eFluor450-conjugated anti-CD45 (mouse monoclonal OX1, Thermo Fisher Scientific), eFluor450-conjugated anti-CD8 (mouse monoclonal OX8, Thermo Fisher Scientific), FITC-conjugated anti-MHCclassII (mouse monoclonal OX17, Biolegend, San Diego, CA), FITC-conjugated anti-CD25 (mouse monoclonal OX39, Biolegend), APC-conjugated anti-CD45RA (mouse monoclonal OX33, Thermo Fisher Scientific), PE-conjugated anti-CD4 (mouse monoclonal OX35, Thermo Fisher Scientific), PE-conjugated anti-CD11bc (mouse monoclonal OX42, Thermo Fisher Scientific), PerCPCy5.5-conjugated anti-CD43 (mouse monoclonal W3/13, Biolegend), PerCPCy5.5-conjugated anti-Foxp3 (rat monoclonal FJK-16 s, Thermo Fisher Scientific), APC-conjugated anti-CD4 (mouse monoclonal OX35, Thermo Fisher Scientific), PECy7-conjugated anti-CD11bc (mouse monoclonal OX42, Biolegend), PECy7-conjugated anti-CD8 (mouse monoclonal OX8, Biolegend), APCCy7-conjugated anti-CD45 (mouse monoclonal OX1, Biolegend), biotin-conjugated anti-CD4 (mouse monoclonal OX35, BD Bioscience) with PE/Cyanine5 Streptavidin (Biolegend). Resting microglia - Mg (CD45^int^CD11bc^+^), CD4 T cells (CD4^+^CD11bc^-^), CD8 T cells (CD8^+^CD11bc^-^), neutrophils - Nf (CD11bc^high^CD43^+^). Gating strategies are provided in the **Supplementary Material** (**Supplementary Figures 1**-**4**).

Fixation/Permeabilization Concentrate and Diluent, Intracellular Fixation & Permeabilization Buffer Set, and Permeabilization Buffer (all from Thermo Fisher Scientific), were employed as appropriate. All stainings were performed at 4°C for 30 minutes. The acquisition of the samples was performed using a CytoFLEX flow cytometer (Beckman Coulter, Indianapolis, IN), and analyzed using CytExpert software (Beckman Coulter). Results of cytofluorimetry are presented as percentage of cells or mfi. Percentages are always within CD45^+^ cells, unless specifically stated.

### 2.7 Reverse transcription−real-time polymerase chain reaction

Total RNA was isolated from cells using a mi-Total RNA Isolation Kit (Metabion, Martinsried, Germany). Reverse transcription was performed with the use of random hexamer primers and MMLV (Moloney Murine Leukemia Virus), according to the manufacturer’s instructions (Fermentas, Vilnius, Lithuania). Prepared cDNAs were amplified by using Maxima SYBR Green/ROX qPCR Master Mix (Fermentas, Vilnius, Lithuania) according to the recommendations of the manufacturer in the QuantStudio 3 (Applied Biosystems, Foster City, CA). Thermocycler conditions comprised an initial step at 50 °C for 5 min, followed by a step at 95 °C for 10 min, and a subsequent two-step PCR program at 95 °C for 15 s and 60 °C for 60 s for 40 cycles. The PCR primers (Metabion, Martinsried, Germany) were as follows: β-actin: 5′-CCA GCG CAG CGA TAT CG-3′; 5′-GCT TCT TTG CAG CTC CTT CGT-3′; Gclc: 5’-CAT CTA CCA CGC AGT CAA GG-3’; 5’-TCA TGA TCG AAG GAC ACC AA-3’; Gclm: 5’-ATG CTC CGT CCT TGG AGT T-3’; 5’-GCT GCT CCA ACT GTG TCT TG-3’; Gsr: 5’- GCT CCA TCT CAG TCC GTT CTT-3’; 5’- GAT TCC TGC AGG CCT TAA CC-3’; Hmox1: 5’-TAA GCT GGT GAT GGC TTC CT-3’; 5’-TCT GCT TGT TGC GCT CTA TC-3’; Nqo1: 5’-GAT CCT GGA AGG ATG GAA GA-3’; 5’-TCT GGT TGT CAG CTG GAA TG-3’; IL-6: 5’-GAG GAT ACC ACT CCC AAC AGA CC-3’; 5’-AAG TGC ATC ATC GTT GTT CAT ACA-3’; TNF: 5’- TCG AGT GAC AAG CCC GTA GC-3’; 5’- CTC AGC CAC TCC AGC TGC TC-3’. PCR product accumulation was detected in real-time and for the analysis of the results QuantStudioTM Design&Analysis Software v1.4.3 (Applied Biosystems, Foster City, CA, USA) was used. Relative mRNA expression is presented as 2^−dCt^, where dCt is the difference between Ct values of a gene of interest and the endogenous control (β-actin).

### 2.8 Statistical analysis

**Figure 1:**
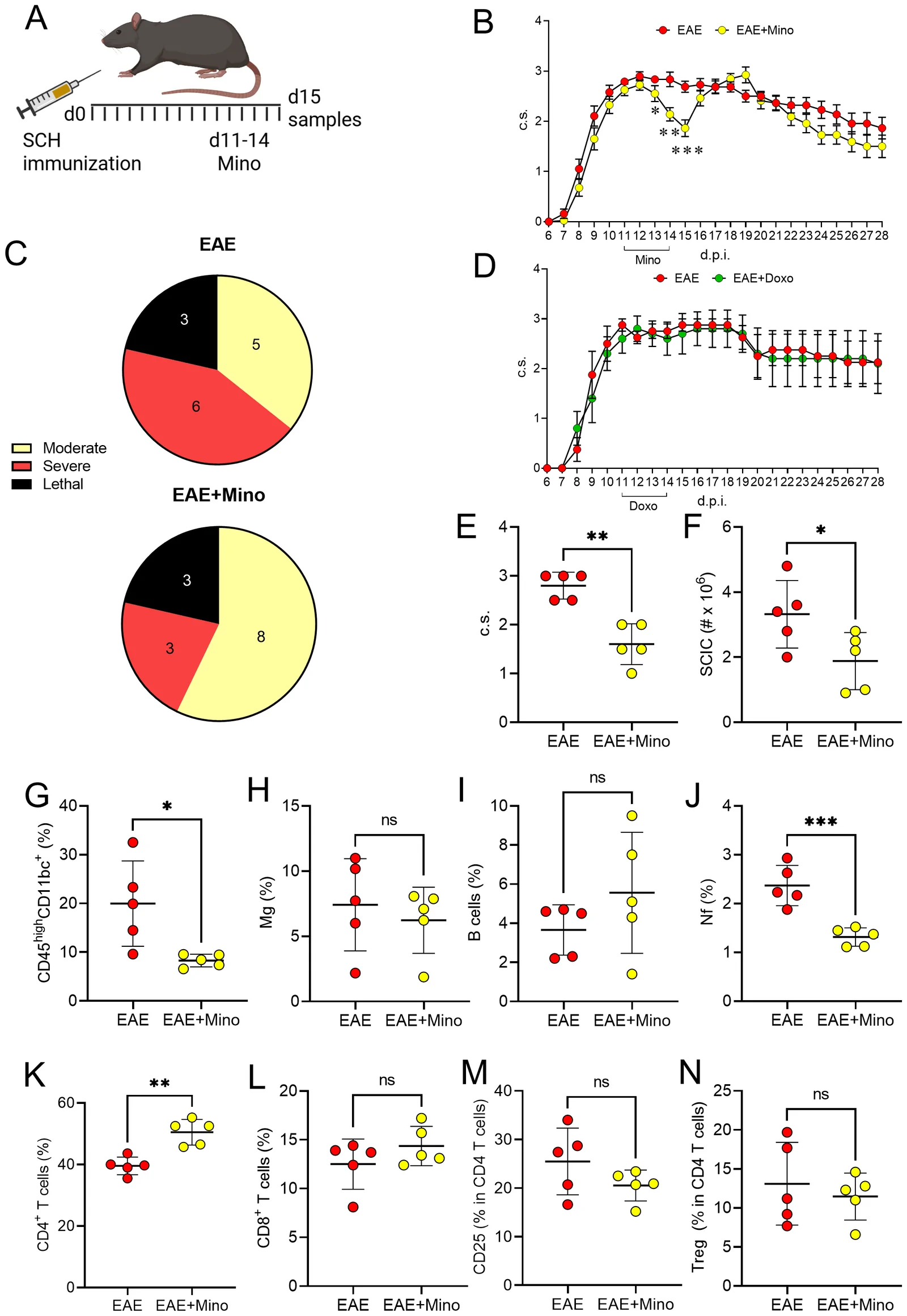
Effect of minocycline administration on EAE. Rats were immunized with SCH, minocycline (EAE+Mino) or vehicle (EAE) were applied to EAE rats from days 11–14 p.i., and spinal cord immune cells were obtained on day 15 p.i. **(A)**. Clinical signs were followed till day 28 p.i. **(B)**. Distribution of rats among different clinical outcomes **(C)**. Clinical signs of rats immunized with SCH, and treated with doxocycline (EAE+Doxo) or vehicle (EAE) from days 11–14 p.i. **(D)**. Clinical score at the time of sacrifice **(E)**. Number of spinal cord immune cells (SCIC) **(F)**. Percentage of various immune populations among the isolated cells as determined by flow cytometry **(G–N)**. Percentages within CD45^+^ cells **(G–L)**, or within CD4^+^ T cells **(M, N)**. Mg – microglia, Nf – neutrophils. Results are presented as mean ± SEM (n=14 in B, C, n=5 in D), or as individual values and mean ± SD from 5 rats **(E–N)**. *p<0.05, **p<0.01, ***p<0.001, ns – not significant, two-tailed Mann-Whitney test **(B, E, I)**, unpaired two-tailed t-test **(F-H, J–N)**.

Sample size estimations were made in accordance with our previous studies, with value for alpha set at 0.05 and power at 0.80 using sample size calculator (University of Vienna, Austria). GraphPad Prism 9 software (GraphPad Software, San Diego, CA) was used for statistical analysis. Data were presented in histograms (mean + S.D.), line graphs (mean ± S.E.M.), box and whisker diagrams (minimum to maximum), and stacked bar charts (mean). Where appropriate, a one-way analysis of variance (ANOVA) with Tukey’s multiple comparison test and a two-way ANOVA with Bonferroni’s multiple comparison test were performed. For comparisons between two groups, Shapiro-Wilk normality test was performed followed by unpaired Student’s t test or nonparametric Mann-Whitney test. P < 0.05 was considered statistically significant. Effect sizes were calculated as Cohen’s d. For most of the statistically significant differences, effect sizes were large (d ≥ 1.12), with the exception in **Figure 1B** d.p.i. 13 and **Figure 5P** EAE wt vs EAE Nrf2^-/-^ where they were moderate (0.50 and 0.55, respectively).

## 3 Results

### 3.1 Minocycline downregulates EAE

To test the effects of minocycline on the ongoing CNS inflammation, EAE rats were treated with minocycline starting at the peak of the disease (**Figure 1A**). This treatment which started at the time of prominent clinical manifestation of the disease (c.s. 2.7 ± 0.4) led to transient reduction of EAE symptoms (**Figure 1B**). Our previous studies have shown that in our DA rat EAE model four clinical courses can be observed, i.e.: a mild, moderate, severe and a lethal course, respectively (19). Importantly, the treatment with minocycline led to a significant shift in the distribution of clinical forms from severe to moderate ones (**Figure 1C**). Thus, besides having immediate effects in reducing of EAE clinical scores, minocycline also had long-lasting effects on the overall clinical course severity. Importantly, another second-generation tetracycline doxocycline applied in the same way as minocycline had no effect on the clinical course of EAE (**Figure 1D**). Namely, cumulative c.s. was 50.1 ± 10.6 *vs*. 48.8 ± 19.1, and maximal c.s. was 2.9 ± 0.3 *vs*. 3.1 ± 0.5 in vehicle- and doxocycline-treated rats, respectively. This result implies that the observed effects of minocycline on EAE are not general feature of second-generation tetracyclines. To examine the effect of minocycline treatment on the autoimmune process, we analyzed immune cells obtained from the inflamed CNS tissue one day after the last minocycline application (**Figure 1A**). At this time point, a clear reduction in clinical score under the influence of minocycline was observed (**Figure 1E**). This clinical effect went along with a significant reduction of the immune cells (**Figure 1F**). Thereby, a relative reduction of CD45^high^CD11bc^+^ cells and neutrophils were observed, whereas the relative numbers of CD4^+^ T cells increased. No effect was found on the numbers of resting microglia, B, and CD8^+^ T cells (**Figures 1 G–L**). Also, there was no change in the proportion of CD25^+^ CD4^+^ T cells and Treg among CD4^+^ T cells under the treatment with minocycline (**Figures 1 M, N**). These results suggest an immunomodulatory shift in immune cell profile in the inflamed CNS tissue of EAE rats under the influence of minocycline.

### 3.2 Minocycline inhibits NO and ROS generation in EAE

**Figure 2:**
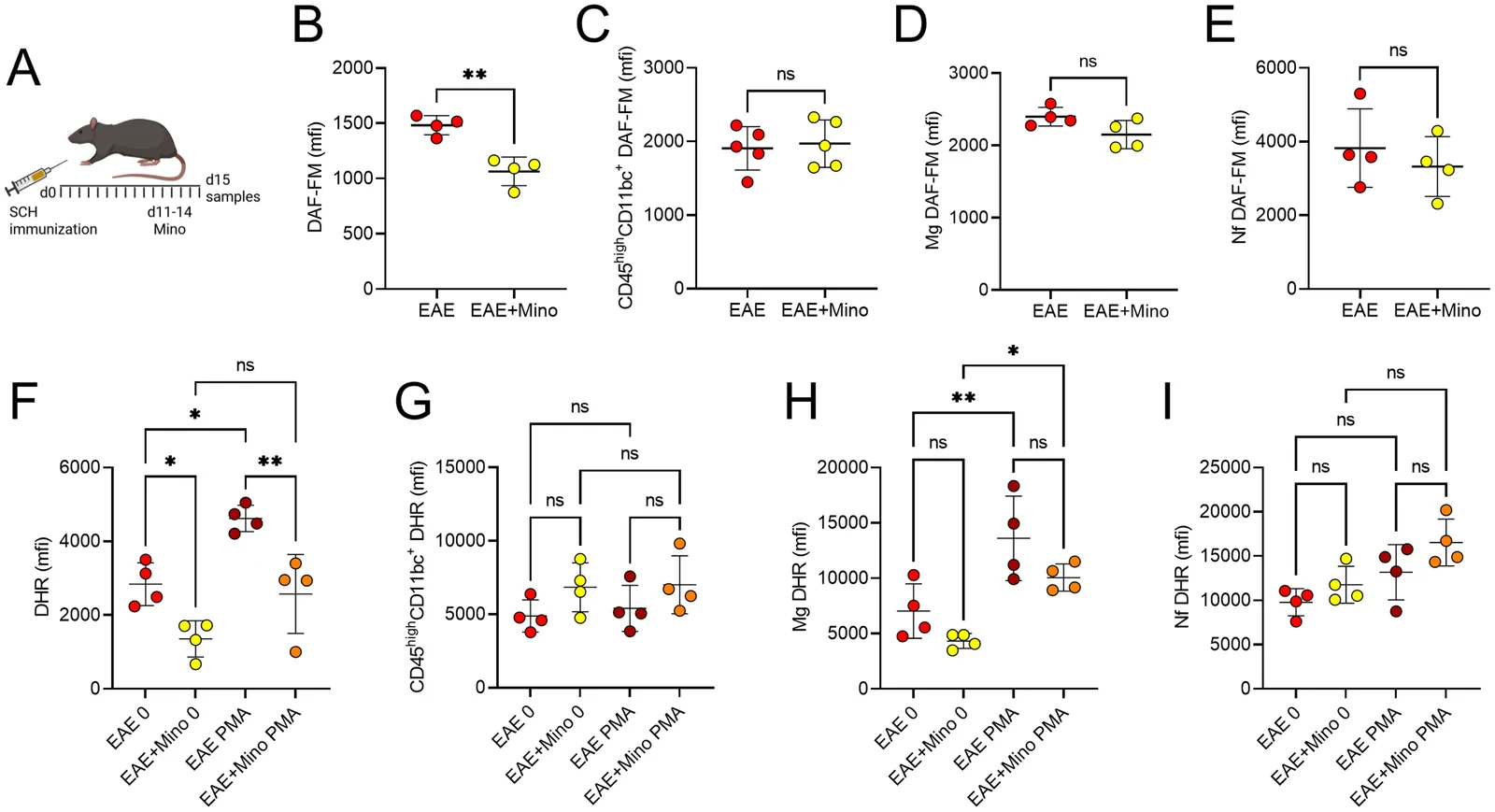
Effect of minocycline on NO and ROS generation in the spinal cord of EAE rats. Minocycline (EAE+Mino) or vehicle (EAE) were applied to EAE rats on days 11–14 p.i. with SCH and the rats were sacrificed on day 15 p.i. **(A)**. NO generation was determined by DAF-FM staining and flow cytometry in unstimulated immune cells isolated from the spinal cord **(B)**, and in CD45^high^CD11bc^+^ cells **(C)**, microglia - Mg **(D)**, and neutrophils - Nf **(E)**. ROS generation was determined by DHR staining and flow cytometry in unstimulated or PMA-stimulated immune cells isolated from the spinal cord **(F)**, CD45^high^CD11bc^+^ cells **(G)**, microglia - Mg **(H)**, and neutrophils - Nf **(I)**. Results are presented as individual values (mfi) and mean ± SD from 4 rats. *p<0.05, **p<0.01, ns – not significant, unpaired two-tailed t-test **(B–E)**, one-way ANOVA followed by Tukey’s multiple comparisons test **(F–I)**.

To explore effects of minocycline on NO and ROS generation by immune cells within CNS in EAE, DAF-FM and DHR staining were performed on immune cells isolated from the spinal cord of EAE rats on day 15 p.i., i.e. one day after the last minocycline administration, when the animals displayed a significant reduction in disease severity (2.8 ± 0.3 vs. 1.6 ± 0.3, p<0.05 t-test; **Figure 2A**). Cells obtained from rats treated with minocycline showed a clear reduction in spontaneous NO production (**Figure 2B**). However, no significant effect on NO production was observed in CD45^high^CD11bc^+^ cells, resting microglia, and neutrophils under the influence of minocycline (**Figures 2C–E**). Further, the production of ROS measured with DHR, either spontaneous ex vivo or PMA-stimulated was downregulated in spinal cord immune cells obtained from minocycline-treated rats (**Figure 2F**). Having in mind that DHR detects not only ROS, but also peroxynitrite, specific measurement of ROS levels was performed with H_2_DCF diacetate, a specific indicator of intracellular ROS. Similar results were obtained using H_2_DCF diacetate, in PMA-stimulated spinal cord immune cells (**Supplementary Figure 5**). Also here, the ROS generation in the analyzed immune cell types, *i.e.* CD45^high^CD11bc^+^ cells, resting microglia, and neutrophils was not significantly reduced under the influence of minocycline (**Figures 2G–I**). Interestingly, resting microglial cells responded to PMA with statistically significant increase in ROS generation (**Figure 2H**), unlike CD45^high^CD11bc^+^ cells (**Figure 2G**) and neutrophils (**Figure 2I**). One likely explanation for the difference in response to PMA might be in the level of activation of the populations within the EAE CNS. While macrophages and neutrophils are highly activated, resting microglia is not. Thus, the lack of response to PMA *ex vivo* might be a consequence of high level of ROS-related activation *in vivo*. Nevertheless, this phenomenon is very interesting and should be explored in details in the future studies.

**Figure 3:**
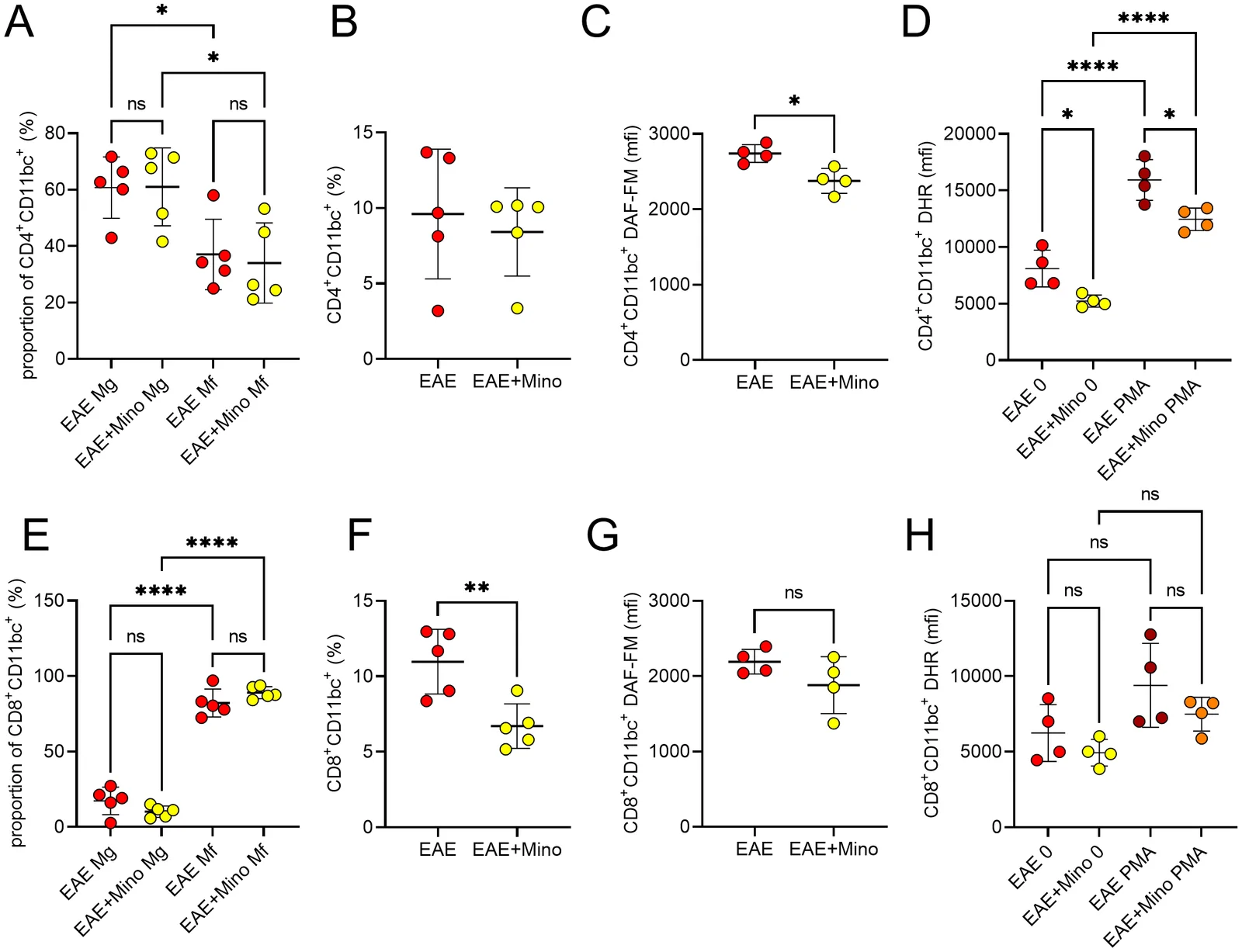
Effect of minocycline on CD4^+^CD11bc^+^ and CD8^+^CD11bc^+^ cells in the spinal cord of EAE rats. Rats were immunized with SCH, minocycline (EAE+Mino) or vehicle (EAE) were applied to EAE rats on days 11–14 p.i. Rats were sacrificed on day 15 p.i. and immune cells isolated from the spinal cord. Proportion of microglial cells (Mg) and macrophages (Mf) among CD4^+^CD11bc^+^ cells **(A)** and CD8^+^CD11bc^+^ cells **(E)**. Proportion of CD4^+^CD11bc^+^ cells **(B)** and CD8^+^CD11bc^+^ cells **(F)** among CD45^+^ cells. NO generation measured by DAF-FM staining and flow cytometry (presented as mfi) in unstimulated CD4^+^CD11bc^+^ cells **(C)** and CD8^+^CD11bc^+^ cells **(G)**. ROS generation measured by DHR staining and flow cytometry (presented as mfi) in unstimulated and PMA-stimulated CD4^+^CD11bc^+^ cells **(D)** and CD8^+^CD11bc^+^ cells **(H)**. Results are presented as individual values and mean ± SD from 4 or 5 rats. *p<0.05, **p<0.01, ****p<0.0001, ns – not significant, two-tailed Mann-Whitney test **(B)**, unpaired two-tailed t-test **(C, F, G)**, one-way ANOVA followed by Šidak’s multiple comparisons test **(A, D, E, H)**.

In order to find out which cell population is responsive to minocycline treatment, CD4^+^CD11bc^+^ and CD8^+^CD11bc^+^ cells that were previously identified as abundant in the spinal cord of EAE rats (18) were examined. Both cell populations consisted of macrophages (CD45^high^CD11bc^+^) and resting microglia (CD45^int^CD11bc^+^), with microglia been more prevalent among CD4^+^CD11bc^+^ cells and macrophages dominantly present among CD8^+^CD11bc^+^ cells (**Figures 3A, E**). Minocycline treatment did not affect the proportion of CD4^+^CD11bc^+^ cells, but it led to the decrease of CD8^+^CD11bc^+^ cell proportion in EAE rats (**Figures 3B, F**). NO and ROS generation was reduced in CD4^+^CD11bc^+^ cells, but not in CD8^+^CD11bc^+^ cells under the influence of minocycline (**Figures 3C, D, G, H**). Specific measurement of ROS levels in CD4^+^CD11bc^+^ cells was performed with H_2_DCF diacetate. CD4^+^CD11bc^+^ cells stimulated with PMA had decreased ROS generation under the influence of minocycline treatment (**Supplementary Figure 5**). To further examine the source of ROS in CD4^+^CD11bc^+^ cells, these cells were treated with acetovanillone, an inhibitor of NADPH oxidase. The inhibitor was efficient in reducing DHR signal (**Supplementary Figure 5**). Thus, NADPH oxidase was, at least partly, responsible for ROS generation in CD4^+^CD11bc^+^ cells in EAE. These results clearly show that both ROS and NO generation in EAE CNS is reduced under minocycline treatment and that CNS CD4^+^CD11bc^+^ cells are the major target in minocycline-imposed restriction of ROS and NO production.

### 3.3 CD4^+^CD11bc^+^ cells abundance in the brain is associated with EAE severity

**Figure 4:**
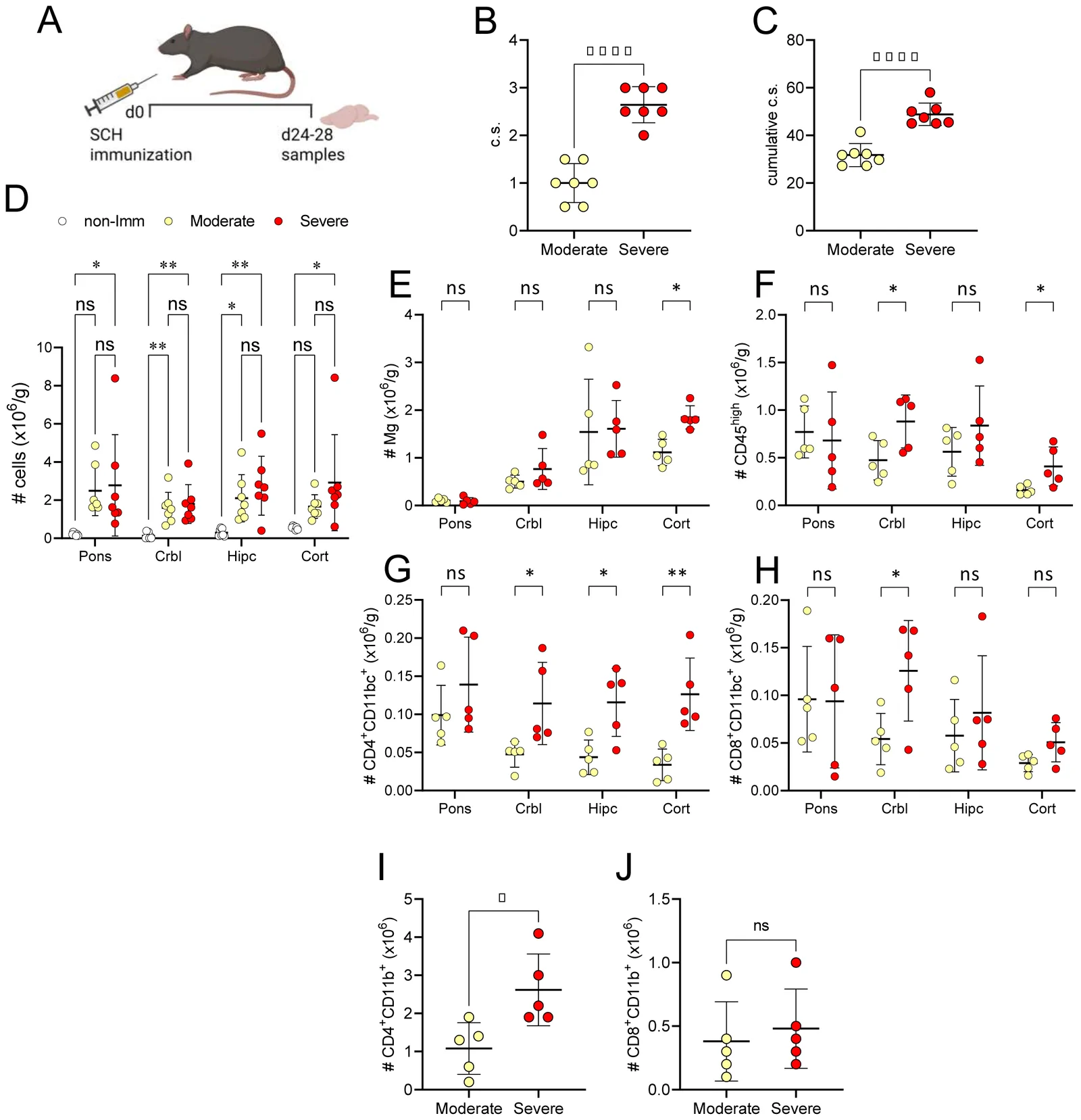
Immune cell profile in the brain at the late phase of EAE. DA rats were immunized with SCH and followed till 24–28 d.p.i. at which time spinal cord and brains were extracted and pons (Pons), cerebellum (Crbl), hippocampus (Hipc), and cortex (Cort) isolated **(A)**. c.s. at the time of sacrifice **(B)** and cumulative c.s. **(C)**. Number of cells obtained from the structures of non-immunized rats (non-Imm), and EAE rats with moderate and severe form of the disease, determined by cell counting **(D)**. Number of microglial cells (Mg), CD45^high^ immune cells, CD4^+^CD11bc^+^ cells, and CD8^+^CD11bc^+^ cells determined as proportion of CD45^+^ cells by flow cytometry and calculated per gram of tissue **(E–H)**. Number of CD4^+^CD11bc^+^ cells **(I)** and of CD8^+^CD11bc^+^ cells **(J)** in spinal cord. Results are presented as individual values and mean ± SD from 7 **(B–D)** or 5 **(E–J)** samples. *p<0.05, **p<0.01, ****p<0.0001, ns – not significant, one way ANOVA followed by Tukey’s multiple comparisons test **(D)** or unpaired two-tailed t-test **(B, C, E–J)**.

As our results so far indicated that CD4^+^CD11bc^+^ cells are the major target of minocycline in EAE, and of importance for the severity of EAE, our next aim was to investigate their abundance in various brain structures, i.e. pons, cerebellum, hippocampus, and cortex in EAE. We focused on late time point (24–28 d.p.i.) when differences between moderate and severe form of the disease are evident both in actual and cumulative c.s. (**Figures 4A–C**). There was a clear increase in the number of immune cells in both moderate and severe EAE in comparison to non-immunized rats, while the numbers were not different between the two EAE groups (**Figure 4D**). The increase of microglial cells was observed only in the cortex of rats with severe EAE in comparison to those with moderate disease (**Figure 4E**). An increase of blood-borne cells was observed in the cerebellum and the cortex (**Figure 4F**). CD4^+^CD11bc^+^ cells were increased in severe EAE in all examined structures but the pons (**Figure 4G**). In parallel, the increase of CD8^+^CD11bc^+^ cells was observed in cerebellum only (**Figure 4H**). Increased abundance of CD4^+^CD11bc^+^ cells (**Figure 4I**), but not of CD8^+^CD11bc^+^ cells (**Figure 4J**) was also observed in spinal cord of severe EAE rats. Thus, infiltration of CD4^+^CD11bc^+^ cells in the CNS was prominently associated with EAE severity.

### 3.4 Minocycline inhibits generation of NO and ROS and ameliorates EAE in Nrf2-independent way

**Figure 5:**
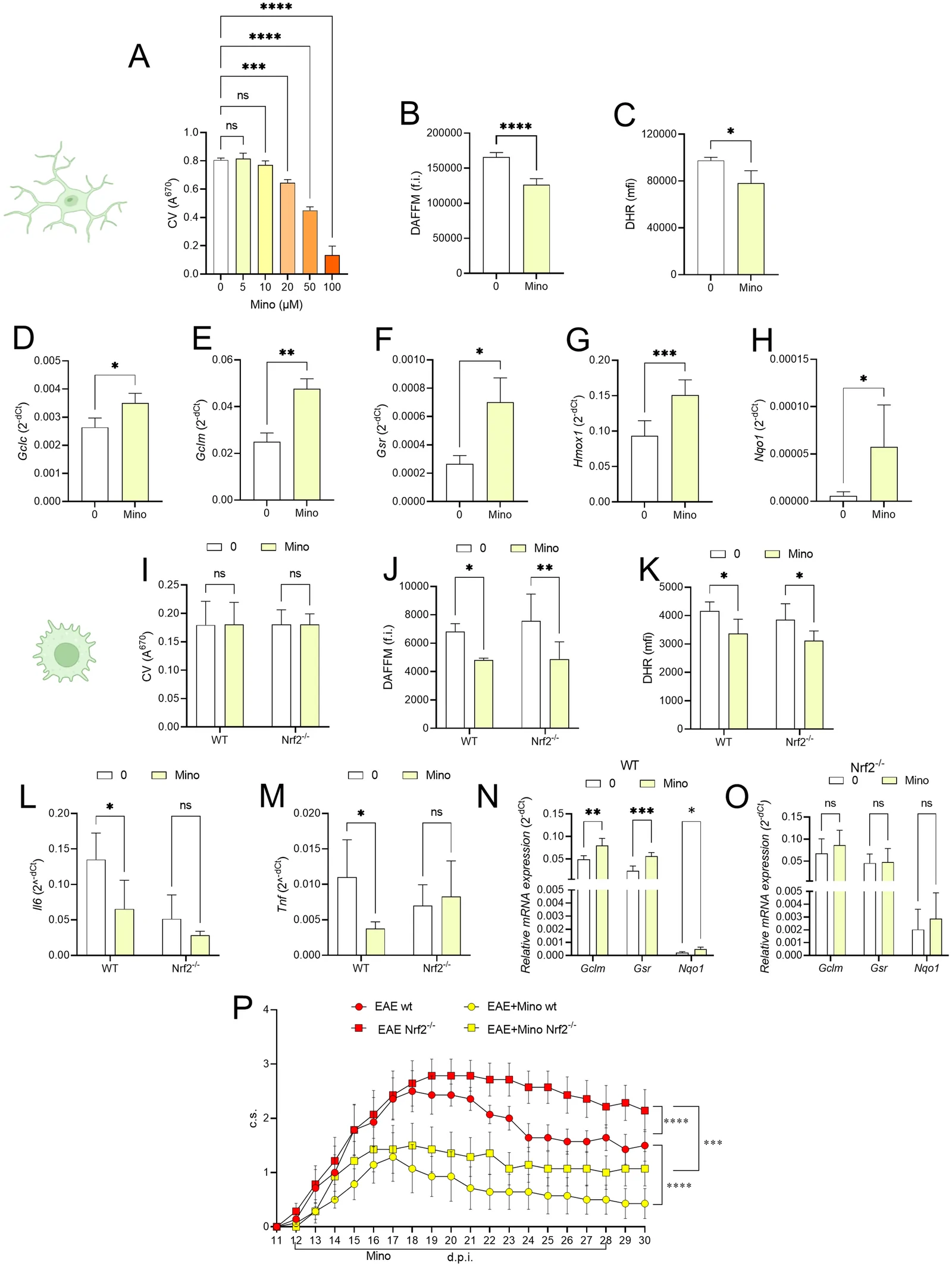
Effects of minocycline on NO and ROS generation *in vitro* and on EAE in Nrf2^-/-^ mice. BV2 cells **(A–H)** and peritoneal macrophages obtained from WT and Nrf2-/- mice **(I–O)** were stimulated with IFN-γ+LPS or LPS, respectively, in the absence (0) or presence of 5 µM minocycline (Mino) for 24 hours **(A–C, I–K)** or 4 hours **(D–H, L–O)**. Afterwards cell viability was determined by crystal violet (CV) test **(A, I)**, ROS and NO generation were determined by DAF-FM diacetate **(B, J)** and DHR **(C, K)** staining, respectively, and relative mRNA expression was measured by real time RT-PCR **(D–H, L–O)**. EAE was induced in WT and Nrf2^-/-^ mice. Minocycline (EAE+Mino) or vehicle (EAE) were applied to EAE mice from 11–28 day p.i. Clinical signs were followed till day 30 p.i. **(P)**. Data from 3 samples are presented as mean + SD **(A-O)** or from 7 mice per group **(P)**. *p<0.05, **p<0.01, ***p<0.001, ****p<0.0001, ns – not significant, one-way ANOVA followed by Dunnett’s test **(A)**, unpaired two-tailed t-test **(B, C, E, G, H, N, O)**, one-tailed Mann-Whitney test **(B)**, two-way ANOVA followed by Šidak’s multiple comparisons test **(I–M)** or Tukey’s multiple comparisons test **(P)**.

To confirm the effects of minocycline on NO generation, BV2 cells or primary macrophages were stimulated *in vitro* with IFN-γ and/or LPS in the presence or absence of minocycline. In fact, minocycline applied in concentrations that did not significantly affect cell viability (**Figure 5A**) potently inhibited NO and ROS production in the BV2 cells (**Figures 5B, C**). We next tested if minocycline regulates Nrf2 activity. To this end we determined the expression of the Nrf2-regulated genes *Gclc*, *Gclm*, *Gsr*, *Hmox1*, and *Nqo1* in minocycline-treated BV2 cells (**Figures 5D–H**). Indeed, there was a clear significant upregulation of these genes following the treatment with minocycline. In order to explore if Nrf2 activation is involved in the observed inhibitory effects of minocycline on ROS and NO generation, peritoneal macrophages obtained from Nrf2^-/-^ mice and their WT counterparts were stimulated with LPS in the presence of minocycline. Surprisingly, minocycline efficiently inhibited NO and ROS generation both in macrophages of WT as well as Nrf2^-/-^ animals (**Figures 5I–K**). However, the inhibitory effect of minocycline on *Il6* and *Tnf* expression was observed in macrophages obtained from WT, but not in those from Nrf2^-/-^ mice (**Figures 5L, M**). Further, minocycline was able to upregulate expression of *Gclm*, *Gsr*, and *Nqo1* in macrophages obtained from WT, but not from Nrf2^-/-^ mice (**Figures 5N, O**), thus implying that minocycline cannot stimulate expression of classical Nrf2-downstream genes in Nrf2-independent way. Taken together, these results imply that minocycline is able to activate Nrf2, but that its effects on ROS and NO generation in macrophages are independent of Nrf2-activation.

Our *in vitro* results thus imply that the effects of minocycline on macrophages/microglia ability to generate NO and ROS are Nrf2-independent. As NO and ROS production reduction in CNS macrophages/microglia was associated with amelioration of EAE severity under the influence of minocycline, we next examined if Nrf2 activation is necessary for the effects of the drug on EAE. Thus, WT and Nrf2^-/-^ mice were immunized and treated in parallel with the compound starting with the appearance of the first clinical signs. Immunization induced more severe EAE in Nrf2^-/-^ mice than in WT mice, while minocycline was equally efficient in ameliorating EAE in WT and Nrf^-/-^ mice (**Figure 5P**). These results therefore show that Nrf2 activation is not essential for the therapeutic effect of minocycline on EAE.

## 4 Discussion

This study demonstrates that minocycline efficiently ameliorates ongoing CNS autoimmunity in EAE. The effect is associated with a reduced ability of CNS CD4^+^CD11bc^+^ cells to generate NO and ROS within the CNS. Interestingly, minocycline anti-encephalitogenic effects are Nrf2-independent. CD4^+^CD11bc^+^ cell abundance in the brain is associated with the severe form of EAE.

Minocycline has been used in EAE and other neuroinflammatory animal models with a considerable efficacy (2–4, 22–29). In previous EAE studies, minocycline was applied either from the day of immunization, or at the appearance of first clinical signs (2, 4, 22–25). In our study, minocycline was applied only at the peak of EAE to mimic treatment of multiple sclerosis patients with severe neurological deficits. Importantly, minocycline was efficient in reducing clinical scores in rats suffering from paralysis, implying that this drug might be a viable solution for multiple sclerosis patients as well. Although the immediate effect was lost after the cessation of minocycline application, long-lasting effects on severity of the disease course were evident. Thus, our results show for the first time that minocycline treatment is efficient in counteracting fully developed CNS inflammation. Doxycycline, another representative of second generation tetracyclines, administered to rats in the same way as minocycline, had no effect on EAE severity or course. This implies that anti-encephalitogenic effects could be specific for minocycline, and not tetracyclines in general. The difference in the anti-encephalitogenic efficacy of the two structurally similar compounds, might be explained by the known fact that minocycline, but not doxycycline readily crosses blood-brain-barrier (30, 31). Importantly, the efficiency of minocycline on full-blown inflammation in EAE suggests that this drug might be beneficial in humans, as well. Still, having in mind the differences in the clinical course and pathogenic properties between EAE and the disease in humans, it will be a challenging step to design possible therapeutic regimens that would be efficient in the treatment of multiple sclerosis patients.

Minocycline is generally considered an inhibitor of microglial activity. Indeed, it was shown to suppress NADPH oxidase-derived ROS production and expression of inducible NO synthase and IL-1β by thrombin-induced microglial cells *in vivo* (32). It was also shown to inhibit proliferation of microglial cells in LPS-induced neuroinflammation and EAE (3). Further, minocycline inhibited phagocytosis and expression of disease-associated genes in a model of photoreceptor cell degeneration (33). Interestingly, minocycline protected microglial cells from ferroptosis during cerebral ischemia-reperfusion injury (34). Finally, minocycline inhibited pro-inflammatory activation, promoted autophagy, and restored mitochondrial function in microglial cells in sepsis-induced neuroinflammation (35). However, it is clear that minocycline affects other cell populations besides microglia in the inflamed CNS, including macrophages. *In vitro* studies convincingly show anti-inflammatory effect of minocycline on monocytes/macrophages, as minocycline was shown to inhibit MHC class II expression, NO production, inflammatory cytokine release in monocytes/macrophages (36–39). Still, studies exploring the effect of minocycline *in vivo* in the context of neuroinflammation are scarce. For instance, it was reported that minocycline reduced number of macrophages in the brain of Theiler’s murine encephalitis virus-infected mice (40). In our study we clearly show that administration of minocycline to EAE rats affects both microglia and CNS macrophages. Moreover, we show that CD4^+^CD11bc^+^ cells, consisted of both microglial cells and macrophages are the dominant target of minocycline in the CNS of EAE rats. In our previous paper, it is shown that CD4^+^CD11bc^+^ cells are numerous and that the level of expression of MHC class II molecules on these cells is high at the peak of EAE in spinal cord homogenate-immunized DA rats (18). Similar results were previously reported in MBP-immunized EAE Lewis rats (15). Further, CD4^+^CD11bc^+^ cells were also abundant in the spinal cord in late phase of EAE, i.e. on 24–28 d.p.i. in our previous study (19). Here, we show that their higher abundance in the spinal cord, the cerebellum, the hippocampus and the cortex is associated with the severe form of EAE. All of these results imply that the activity of CD4^+^CD11bc^+^ cells is significant for the clinical outcome of EAE. Association of EAE restriction by minocycline with the limitation of the ability of CNS CD4^+^CD11bc^+^ cells to generate ROS and NO observed in this study implies that this population of CNS cells have a deleterious role in EAE. Although minocycline did not restrict ability of CNS CD8^+^CD11bc^+^ cells to produce ROS and NO, it did reduce their proportion within spinal cord immune cells, thus suggesting that further investigation of the effect of minocycline on CD8^+^CD11bc^+^ cells in EAE is warranted.

ROS and NO are the major effector molecules in EAE and multiple sclerosis pathogenesis (41). Peroxynitrite, a product of superoxide and NO, is especially deleterious molecule in the CNS autoimmunity as it is highly toxic to oligodendrocytes (42). Moreover, peroxynitrite is also able to interfere with regulation of autoimmune response, as nitration of IL-2R leads to functional reduction of Treg (43). Thus, the ability of minocycline to inhibit release of both ROS and NO in inflamed CNS is highly relevant for its potential use in the treatment of multiple sclerosis.

Minocycline was shown to limit nephropathy in Nrf2-dependent way in diabetic mice (13). To the best of our knowledge, this is the only study in which minocycline dependence on Nrf2 was shown using Nrf2^-/-^ mice. Further, there were studies in which dependence of minocycline neuroprotective effects on Nrf2 was confirmed using small interfering RNA *in vitro* (14, 44). However, the effects of minocycline on neuroinflammation that were previously associated with its ability to potentiate Nrf2 activation (11, 12) were without direct proofs of Nrf2-dependent action in neuroinflammation. In our study, we used Nrf2^-/-^ mice for the first time in the context of neuroinflammation, i.e. in EAE. Notably, minocycline was equally efficient in ameliorating EAE in wild type and Nrf2^-/-^ mice, and we can confidently claim that minocycline effects on neuroinflammation in this setting is Nrf2-independent. Also, it was shown that minocycline imposed inhibition of ROS production in neuroglial cells is Nrf2-dependent (14), but our results show that ROS and NO production in macrophages is Nrf2-independent. Thus, it seems that Nrf2 is important, but not necessary for antioxidative effects of minocycline in neuroinflammation. Interestingly, the effect on pro-inflammatory cytokine expression in macrophages is Nrf2-dependent in our system. This implies that minocycline limits detrimental effects of ROS and NO in the CNS in an Nrf2-independent manner, while Nrf2 might still be relevant for the regulation of other neuroinflammatory processes by minocycline. Also, previous studies identified various mechanisms behind minocyline beneficial effects in EAE. For instance, minocycline was shown to inhibit extracellular matrix metalloproteinase inducer and matrix metalloproteinases 2 and 9 on immune cells, and to increase tissue inhibitors of metalloproteinases, brain-derived neurotrophic factor, and nerve growth factor (2, 23–25, 45). Further studies are needed to determine if these and other mechanisms behind minocycline beneficial effects in EAE are Nrf2-dependent or not. Finally, further search for the essential pathways behind the anti-encephalitogenic effects of minocycline is necessary.

In conclusion, our results support the view that minocycline is a potent immunomodulatory agent with valuable effects in CNS autoimmunity. Importantly, our results identify CNS CD4^+^CD11bc^+^ cells as the target of minocycline in rat EAE. Although it is known that human macrophages/microglia express CD4, we are not aware of studies on CD4^+^CD11bc^+^ CNS cells in the context of multiple sclerosis. Such studies will be needed to understand if CD4^+^CD11bc^+^ CNS cells contribute to the pathogenesis of multiple sclerosis, as well as to explore the possibility to target CD4^+^CD11bc^+^ cells in the therapy of the disease. Thus, studies in which CD4^+^CD11bc^+^ cells would be identified in the brain lesions and/or cerebrospinal fluid of MS patients are urgently needed. Following their identification thorough characterization of their genomic and proteomic profile, along with functional tests will be required. Our results suggest that such a direction in multiple sclerosis research is necessary as it might contribute to our understanding of the disease pathogenesis and minocycline therapeutic opportunities.

## Data Availability

The original contributions presented in the study are included in the article/**Supplementary Material**. Further inquiries can be directed to the corresponding author.
