# Supplementary material for: Minocycline ameliorates CNS autoimmunity through restraint of CD4+CD11bc+ cells
Source: Front Immunol. 2026 Sep 3;17:1823730. doi: 10.3389/fimmu.2026.1823730 (PMC13582497; doi:10.3389/fimmu.2026.1823730)
Supplement: Supplementary file 1 [file SupplementaryFile1.pdf]

Supplementary Material

1 Supplementary Figures

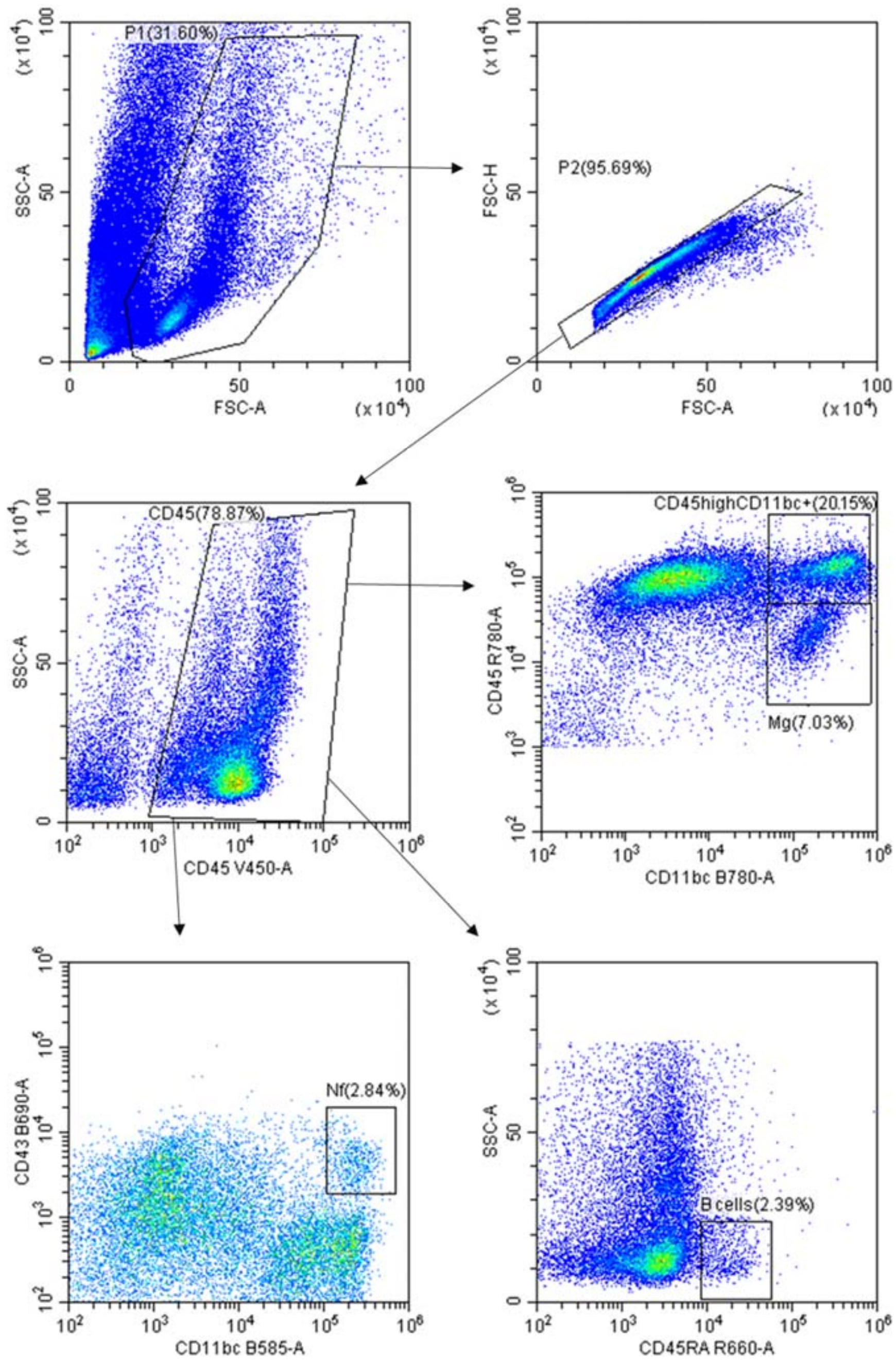

Supplementary Figure 1. Gating strategy for microglial cells, neutrophils and B cells. Cells (P1) were gated to singlets (P2) and then to CD45^{+}. CD45^{high}CD11bc^{+}, microglial cells (Mg, CD45^{low}CD11bc^{+}), neutrophils (Nf, CD11bc^{high}CD43^{+}) and B cells (CD45RA^{+}) were determined among CD45^{+}cells.

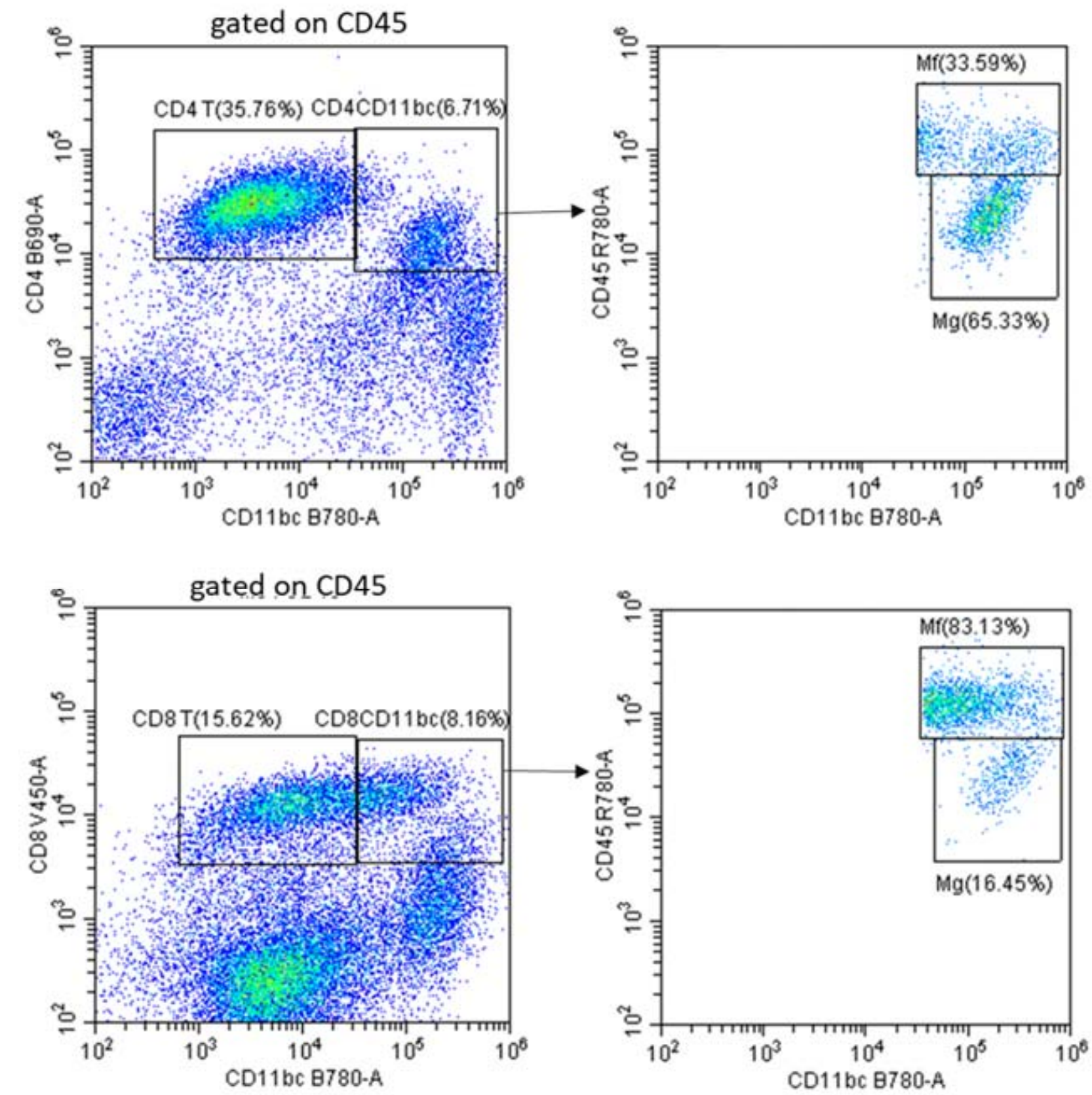

Supplementary Figure 2. Gating strategy for CD4$^+$CD11bc$^+$ and CD8$^+$CD11bc$^+$. Cells were gated to CD45$^+$ as shown in Suppl Fig 1. CD4$^+$CD11bc$^+$ and CD8$^+$CD11bc$^+$ were determined within CD45$^+$ cells. Macrophages (Mf, CD45^{high}CD11bc$^+$) and microglial cells (Mg, CD45^{low}CD11bc$^+$) were determined among CD4$^+$CD11bc$^+$ and CD8$^+$CD11bc$^+$ cells.

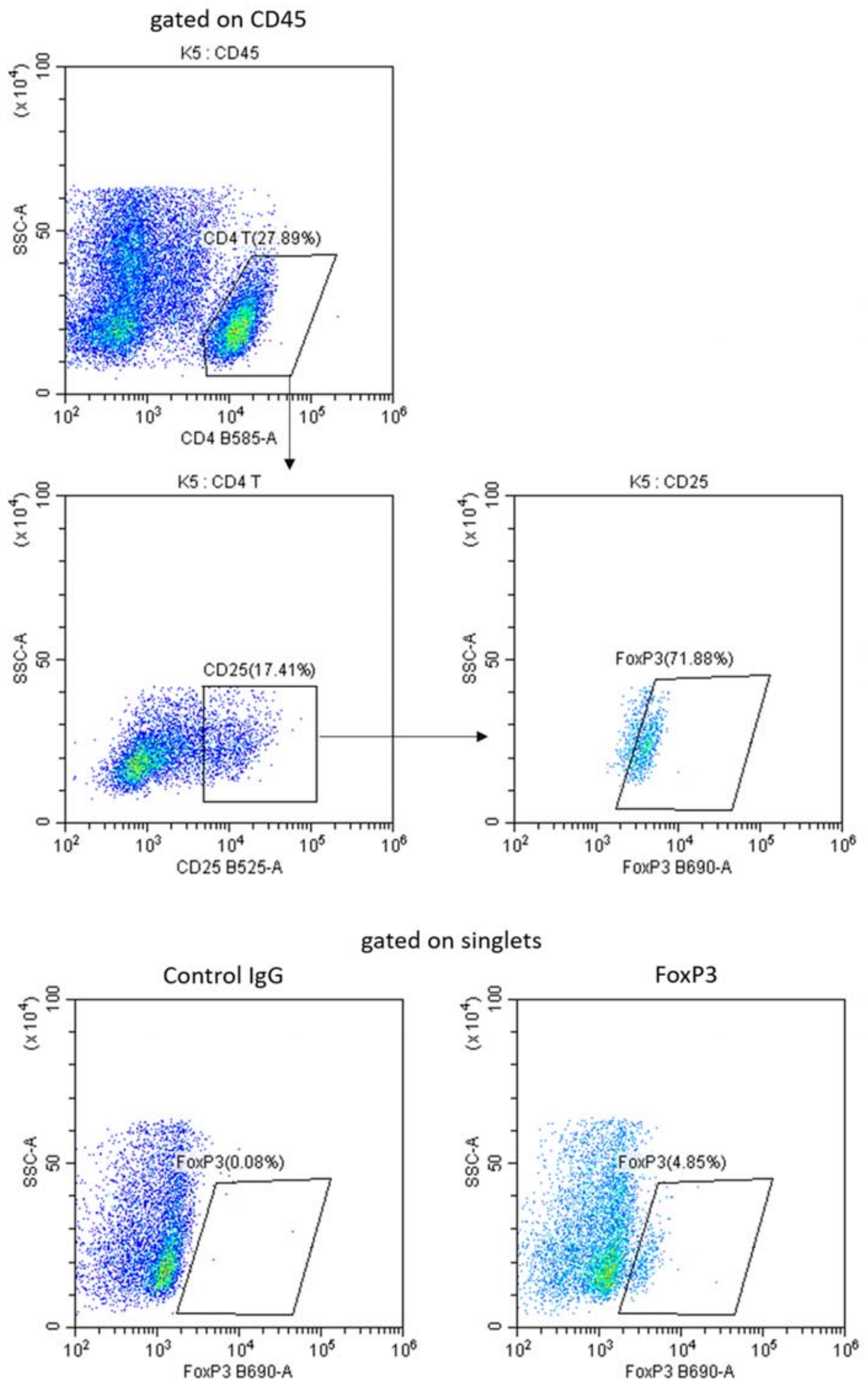

Supplementary Figure 3. Gating strategy for Treg. Cells were gated to CD45^{+} as shown in Suppl Fig 1. Treg were determined as FoxP3^{+} within CD25^{+} within CD4 T. To determine gate for FoxP3, cells were gated to singlets (gate P2 in Fig 1). Irrelevant rat IgG2 kappa antibody and FoxP3 stained cells are shown.

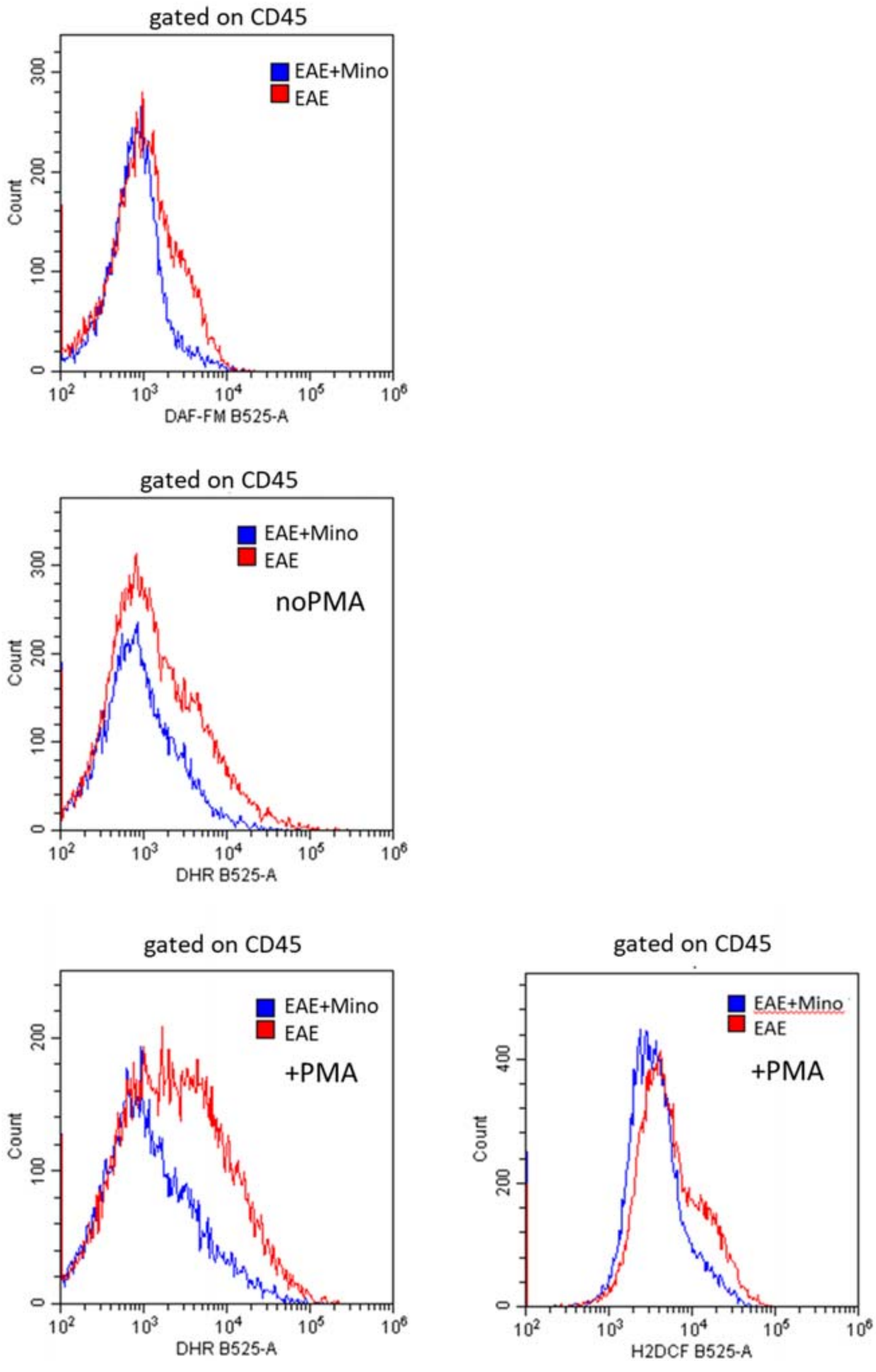

Supplementary Figure 4. Representative plots for DAF-FM, DHR, and H_2DCF staining. Cells were gated to $CD45^+$ as presented in Suppl Fig 1. Upper panel – DAF-FM, middle panel – DHR in non-stimulated cells, lower panel left – DHR in PMA-stimulated cells, lower panel right – H_2DCF in PMA-stimulated cells.

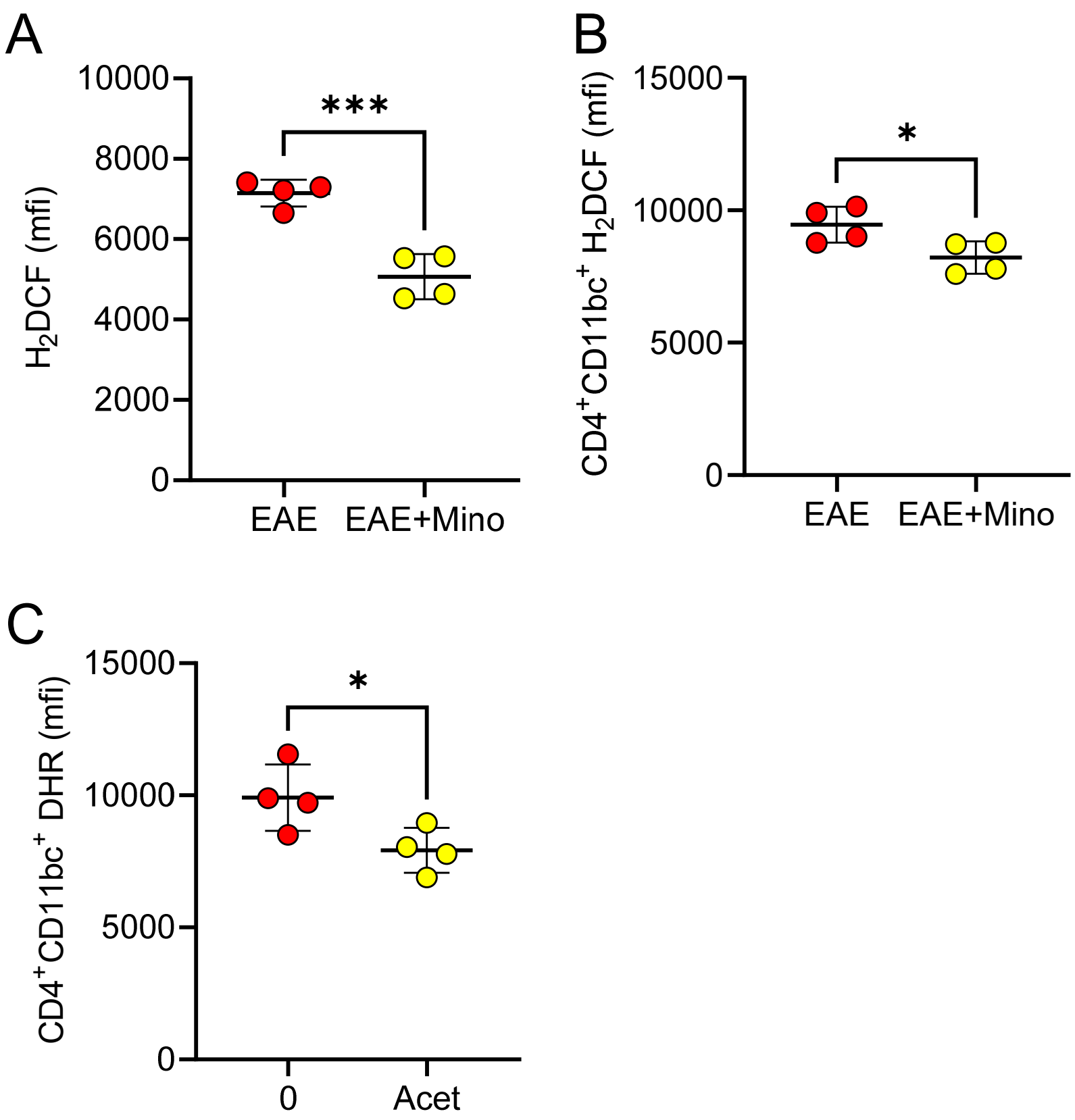

Supplementary Figure 5. Effect of minocycline and acetovanillone on ROS generation in spinal cord immune cells of EAE rats. Rats were immunized with SCH, minocycline (EAE+Mino) or vehicle (EAE) were applied to EAE rats on days 11-14 p.i. Rats were sacrificed on day 15 p.i. and immune cells isolated from the spinal cord. Intracellular ROS was measured by H_2DCF diacetate staining in total cells (A) and $CD4^+CD11bc^+$ cells (B). Spinal cord immune cells from EAE rats were treated with acetovanillone (Acet, 100 µM) and DHR staining was performed (C). Results are presented as individual values (mfi) and mean ± SD from 4 rats. $^*p<0.05$, $^{***}p<0.001$, ns – not significant, unpaired t-test.
